# Perspective: Potatoes, Quality Carbohydrates, and Dietary Patterns

**DOI:** 10.1016/j.advnut.2023.10.010

**Published:** 2023-10-30

**Authors:** Stephen A. Fleming, Jenny R. Morris

**Affiliations:** Traverse Science, Mundelein, IL, United States

**Keywords:** potatoes, carbohydrate quality, epidemiology, dietary patterns, type 2 diabetes, nutrient profiling

## Abstract

Potatoes have long been a staple food in many cultures and cuisines, but they have gained a reputation as a low-quality carbohydrate source that should be avoided in the diet. Historically, this view has been justified by citing the glycemic index of potatoes as the main indicator of their quality. However, their nutrient composition should also be considered. The association of potatoes with energy-dense Western dietary patterns has also contributed to a perception that potatoes are inherently unhealthy. Although some studies have suggested an association between potato consumption and increased risk of health problems, such as type 2 diabetes, these associations may be confounded by fried potato intake and are strongest at intake levels higher than average consumption rates. Epidemiologic data suggest total potato intake is not a health risk in Eastern populations and can be consumed as part of a healthy diet. Furthermore, clinical trial data demonstrate that potatoes’ health impact, irrespective of preparation, is similar to legumes and comparable with refined grains, with few deleterious effects found. These findings highlight the importance of moving beyond the glycemic index and adopting a more nuanced evaluation of the epidemiologic data to better understand the health impact of potato intake. Ultimately, the negative reputation of potatoes stems from an overinterpretation of their glycemic index and association with unhealthy Western dietary patterns, as well as oversimplification of the epidemiologic data. By considering carbohydrate quality, it becomes clear that potatoes can be part of a healthy diet given the proper consideration.

**Statement of Significance**

This comprehensive perspective of the nutritional quality and health impact of potatoes moves beyond the oversimplified glycemic index-based view of their carbohydrate content. By contextualizing the epidemiologic data and examining clinical trial evidence, this work offers a more nuanced evaluation on the role of potatoes in a healthy diet, which may inform dietary recommendations and improve public health outcomes.

## Introduction

The debate on the role of the potato in the diet has spanned decades. In 1999, Dr Gilbert Forbes wrote a letter to the editor in The American Journal of Clinical Nutrition addressing Dr Walter Willett’s placement of potatoes at the top of the United States food pyramid [1]. Forbes [1] noted that this position afforded the potato “second class” status and contended that potatoes should be moved toward similar foods, such as wheat and rice. Dr Willett noted the potato’s propensity to increase the risk of type 2 diabetes mellitus (T2DM) and its high glycemic index (GI), stating that it deserved its status next to sweets and should be consumed sparingly [1]. This conversation has largely not evolved after 20 y of further study. Arguments promoting potato intake cite the potatoes’ overall favorable nutrient composition [2], whereas evidence from epidemiologic studies highlight greater risk of chronic disease with increased potato intake [3] and the prevalence of potato in the energy-dense Western dietary pattern [4]. We propose that the shadow cast by potatoes’ association with unhealthy dietary patterns, high GI, and increased risk of metabolic disease has obscured the reality of their status as a high-quality carbohydrate food, likeness to other nutrient-dense foods, and the lack of clinical trials to support the epidemiologic data. Here, we discuss why potatoes have received an undeserved reputation based on the overreliance of using the GI as a marker of healthy foods, exaggerated interpretation of the epidemiologic data, and overinterpretation of Western dietary patterns and consumption of fried potatoes.

## Potatoes Are a Nutrient-Dense Vegetable

Potatoes are one of the most sustainable, affordable, accessible, and widely grown crops worldwide [5]. Potatoes are nutrient-rich, particularly in fiber and potassium, and their inclusion in some dietary patterns is associated with higher diet quality compared with their absence in children, adolescents, and adults [6,7]. A baked white potato with the flesh and skin (USDA FoodData Central ID 170434) contains (per 100 g) 92 kcal, 21 g carbohydrate, 2.1 g protein, 0.15 g fat, 2.1 g fiber, 544 mg potassium, and 7 mg sodium [8]. Potatoes score well on carbohydrate quality metrics, similar to legumes and even exceeding that of whole grains in some cases [2]. However, they have developed a reputation as a low-quality carbohydrate [9], with many reviews citing their GI as the cause [3,10,11]. Despite limited published epidemiologic evidence on its potential risk of T2DM until after the 2000s [[12], [13], [14], [15], [16]], the potato was already considered a health concern based on its GI as early as the 1980s [17]. Such early observations have led to the perception that the potato may not belong in healthy dietary patterns. A 2018 meta-analysis revealed that potato consumption is not associated (nonsignificant) with the relative risk (RR) of all-cause mortality (RR: 0.88; 95% confidence interval [CI]: 0.69, 1.12), coronary heart disease (RR: 1.03; 95% CI: 0.96, 1.09), stroke (RR: 0.98; 95% CI: 0.93, 1.03), or colorectal cancer (RR: 1.05; 95% CI: 0.92, 1.20) and is positively associated (statistically significant) with the risk of T2DM (RR: 1.66; 95% CI: 1.43, 1.94) and hypertension (RR: 1.37; 95% CI: 1.15, 1.63) [3]. What is still unclear is whether this relationship indicates a meaningful biological relationship between the nutritional value of potatoes and chronic disease or the manifestation of the overall dietary pattern.

Numerous studies group potatoes with energy-dense Western dietary patterns, giving the potato a “guilty by association” status [4,[18], [19], [20], [21], [22]]. Such groupings have emerged from a posteriori methods (e.g., principle component or factor analysis) demonstrating potatoes are frequently consumed with red meat, butter, eggs, sugar-sweetened beverages, sweets, and other energy-dense foods in Chinese [18], American [4], Greek [19], Canadian [20], British [21], and Iranian [22] populations. With such common findings between studies and across different cultures, it is tempting to conclude that the potato is not only “guilty by association” but that its consumption may be a driver of adverse health. Nevertheless, potatoes have still been found, using a posteriori methods, to be consumed in nutrient-rich dietary patterns containing foods such as pulses, mushrooms, green and yellow vegetables, fish, and fruits in Japanese populations [[23], [24], [25]]. In the Nordic diet, boiled potatoes are recommended among other traditional foods, including but not limited to fruits and vegetables, almonds, legumes, meat, poultry, low-fat or fermented milk and cheese, fish, eggs, and whole-grain cereals [26]. Thus, potatoes appear to have a place in both healthy and unhealthy dietary patterns.

Given dietary pattern studies are not designed to assess a single food’s health impact, a more objective point-of-view can be found in indices assessing the overall quality of carbohydrate-rich foods. As discussed, most explanations for why the intake of potatoes may be a health concern include its high GI [3,10,11]. Although the GI is a valuable metric for assessing what foods produce lower glycemic responses on average [27], it should be considered alongside other quality metrics. A detailed comparison of the value and issues related to the GI are well described elsewhere [27,28]. The Carbohydrate Quality Index (CQI) is calculated based on the free sugar and fiber content of foods with >40% carbohydrate by dry weight, with the Carbohydrate Food Quality Score (CFQS) additionally taking into account the potassium, sodium, or whole-grain content of foods [29,30]. The CQI and CFQS use a point system, with foods receiving 1 point if they meet criteria for lower free sugar and sodium or higher fiber and potassium (Table 1). Higher scores indicate greater nutrient richness. For example, the 10:1:1 CQI affords one point each if a food contains equal or >1 g fiber and <1 g free sugars per 10 g carbohydrate, for a total of 2 points (Table 1). Then, the CFQS-5 includes whole grains in addition to the same metrics as the CFQS-4 (Table 1). The CQI and CFQS metrics are intended to align carbohydrate scores with the recommendations from the Dietary Guidelines for Americans for reducing sodium and free sugars and increasing fiber, potassium, and whole-grain intake [29]. Although the CQI and CFQS do not consider energy, fat, or protein, they correlate well with other indices of food quality. For instance, they show a strong correlation with the Nutrient-Rich Food 9.3 score, which is calculated on 9 nutrients to encourage (protein, dietary fiber, vitamins A, C, and E, calcium, iron, magnesium, and potassium) and 3 nutrients to limit (saturated fat, sodium, and added or total sugar) [29].

**TABLE 1:** Carbohydrate quality point scoring system^1^

Metric	Carbohydrate Quality Index			Carbohydrate Food Quality Scoring System (CFQS)	
	10:1:1	10:1:2	10:1|1:2^2^	CFQS-4	CFQS-5
≥10 g Fiber Per 100 g carbohydrate	1	1	1	1	1
<10 g Free sugars Per 100 g carbohydrate	1	—	—	1	1
<2 g Free sugars Per 100 g carbohydrate	—	1	—	—	—
<2 g Free sugars Per 1 g fiber	—	—	1	—	—
Sodium <600 mg Per 100 g dry weight	—	—	—	1	1
Potassium >300 mg Per 100 g dry weight	—	—	—	1	1
Whole grains ≥25 g Per 100 g dry weight	—	—	—	—	1
**Total points possible**	**2**	**2**	**2**	**4**	**5**

Abbreviation: CFQS, Carbohydrate Food Quality Scoring System.

1 Metrics adapted from Drewnowski et al. [29] and Campos et al. [30].

2 Equivalent to 10:1|2:1 Carbohydrate Quality Index

**FIGURE 1:**
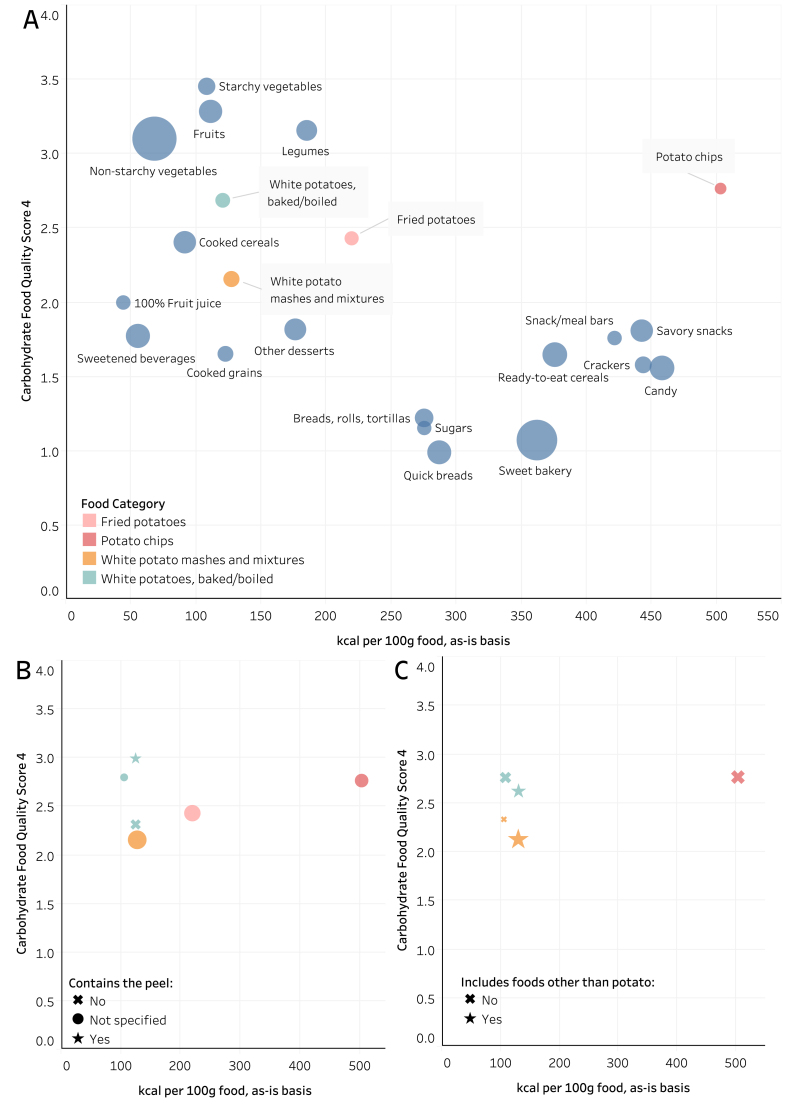
Scatterplots highlighting (A) potato food groups in the Food and Nutrient Database for Dietary Studies, (B) potato foods with or without the peel, and (C) potato food groups with or without other ingredients. Potato categories appeared distinct from refined grains, sweetened beverages, and snacks, with a higher Carbohydrate Food Quality Score 4 when containing the peel. Scores for potato foods with other ingredients were lower than potato alone. Points represent the average of all foods within a category, with the relative size representing the number of foods in that category.

Drewnowski et al. [2] used the CQI and CFQS with the USDA Food and Nutrient Database for Dietary Studies (FNDDS) to demonstrate that potatoes rank similarly to legumes, with higher carbohydrate quality than whole grains and lower energy density. Here, we reproduced the analysis of Drewnowski et al. [2] and further segmented potato foods by their type, presence of the peel, and consumption with other foods (Figure 1). Given the FNDDS does not use the term “free sugars,” these were defined the same as Drewnoski et al. [2] as added sugars; sugars from 100% fruit juice; sugars in sweetened beverages, jams, and jellies; and honey, sugars, and syrups. Although potatoes may be consumed with low-quality carbohydrate sources, their average CFQS-4 score (2.2–2.8) is higher than the average scores for cooked grains, breads, candy, 100% fruit juice, ready-to-eat cereals, and sweetened beverages (Figure 1), each of which have scores ranging between 1 and 2. Baked or boiled white potatoes have the highest CFQS-4 score and lowest energy density, ranking more closely to legumes, fruits, and nonstarchy vegetables than refined grains, sweets, snacks, and sweetened beverages. Surprisingly, the potato chips food group demonstrates the highest CFQS-4 score for potatoes but is clearly the most energy dense of any food category (Figure 1A). Potatoes consumed in mixtures (e.g., potato salad, mashed with butter and sour cream, scalloped, etc.) have the lowest average CFQS score of the potato foods, likely due to added sodium and less fiber per 100 g carbohydrate in each food. Baked or boiled white potatoes are the only potato food group where nutrient data existed with or without the peel. Unsurprisingly, potato foods with the peel had the highest average CFQS-4 (∼3) score among potato groups (Figure 1B). Then, many potato foods in the USDA Food and Nutrient Database for Dietary Studies include other ingredients, for example, a baked potato with sour cream. After separating these entries from food groups that only contained potato, it became clear that the other foods (typically butter, sour cream, cheese, or meat) lower the average CFQS-4 score (Figure 1C) and increase energy density. Although potatoes frequently emerge as part of a “Western” diet, these data demonstrate that potatoes are one of the highest quality sources of carbohydrate consumed in a “Western” diet, rather than the driver of low diet quality itself.

## Available Data from Meta-Analyses Are Relevant to Specific Populations but Do Not Generalize Well to Whole Populations

It should first be noted that no meta-analyses have found potato intake increases the risk of mortality [31] or stroke [3], with mixed evidence linking it to cancer [3,32] and hypertension [3]. However, given the potato has largely been faulted for its high GI, results from meta-analyses focused on cohorts with T2DM can be examined as a case study. Here, data suggest that the population (and potentially their overall dietary pattern) and amount of potato consumed are key to understanding the relationship between potato intake and health.

Four recent meta-analyses of prospective cohorts have described how potato intake increases the risk of T2DM [3,10,33,34]. With such agreement, the hypothesis appears sufficiently answered. Of the 4, Schwingshackl et al. [3], Bidel et al. [33], and Zhang et al. [34] all analyzed the same cohorts from Australia [35], Finland [14], and the United States [12,15], with Schwingshackl et al. [3] additionally including a German cohort [13], and with each adjusting for a variety of additional factors, such as lifestyle, diet, age, weight, and more. The congruence of their findings is indicative of repeat analyses on the same data, and residual confounds may still exist. Guo et al. [10] includes these cohorts in addition to cohorts in central and northern China [36,37], an Iranian cohort [38] and cross-sectional study [39], case-control study in Saudi Arabia [40], and cross-sectional study in the United Kingdom [16], representing the most encompassing meta-analysis across geographies and study designs. Their meta-analysis additionally included gestational diabetes as an outcome [10], but the data discussed here are focused on T2DM. Guo et al. [10] provide the only visualization of intake from individual studies, which revealed the striking pattern that all studies reporting total potato intake >100 g/d demonstrated an increased risk of T2DM, whereas intakes <100 g/d more frequently showed a neutral or even positive impact on the risk of T2DM. Simultaneously, Guo et al. [10] found that total potato intake did not increase the risk of T2DM (RR: 0.94; 95% CI: 0.71, 1.25) in Eastern populations (defined as both Middle East and East Asian populations) as it did (RR: 1.19; 95% CI: 1.06, 1.34) in Western populations (defined as Russia, Europe, the Americas, Australia, and New Zealand). The authors present 2 straightforward but intriguing explanations. Their first point is that Eastern populations in their meta-analysis consumed less potato than Western populations (43.5 g/d compared with 100.8 g/d), and a dose-response effect could explain the differences in geography. The second is that dietary patterns might mask the effects of potato intake in Eastern populations with high refined grain intake, a staple food in Eastern populations. This may be true, but refined grain intake is also high in America, contributing over 6 times the amount of energy to the American diet compared with potatoes [9]. Guo et al. [10] also found that Eastern populations have lower risk of T2DM than Western when consuming fried (Eastern: RR: 1.07; 95% CI: 0.25, 4.55; Western: RR: 1.33; 95% CI: 1.03, 1.70) and baked/boiled/mashed forms (Eastern: RR: 0.84; 95% CI: 0.29, 2.40; Western: RR: 1.08; 95% CI: 1.00, 1.16); however, only 1 to 2 articles each were available to compare Eastern and Western populations on each form, with low precision and high heterogeneity in the Eastern populations. It should also be noted that the studies on Eastern populations were more heterogenous in study design than those on Western populations, which may affect the precision and accuracy of estimates. Other factors, such as differences in energy intake may be at play, but we are aware of no available meta-analysis on the topic designed to take this into account. Regardless, the sentiment that other dietary factors may be responsible for the presence or lack of a relationship between potato intake and health reinforces the idea that the overall dietary pattern, and not the potato itself, may be responsible for the effects shown.

### Potato Consumption in Western Prospective Cohorts Does Not Reflect Today’s Dietary Patterns

The geographic differences shown by Guo et al. [10] hint that a combination of the dose and population may be key to understanding potato intake and health. Guo et al. [10] meta-analyzed United States population data from 2 publications comprising 4 prospective cohorts (the Women’s Health Study, Nurses’ Health Study, Nurses’ Health Study II, and the Health Professionals Follow-up Study), which were primarily composed of middle-aged (45+ y of age), female health professionals nearing overweight [12,15]. Other non-United States Western populations from meta-analyses were similar in composition and timeframe [13,14,16,35], suggesting that much of the epidemiologic evidence informing our understanding of potato intake and its impact on health outcomes rests upon homogenous populations, lowering the generalizability of their findings to other populations. In addition to most prospective cohort data collected on homogenous populations, the dietary patterns of these subjects differed from those of today. Notably, most of the dietary data in these cohorts were collected before 2006, when *trans fats* were required to be labeled in the United States, after which *trans fat* intake (particularly from French fries) reduced precipitously [41]. Although *trans fat* intake is typically associated with cardiovascular disease and not T2DM, these populations represent a period in time where the dietary impact of consuming fried foods was distinct from the recent 15 y. Although potato intake has been stable over the past 20 y [9], we speculate that preparation methods for fried potatoes today lead to a healthier and more nutrient-rich fried potato than those used decades ago. Although such cohort data are highly valuable, the generalizability of the data from such homogenous populations collected when *trans fats* were commonplace in the diet warrants attention.

Beyond the population differences over time, the intakes reported in meta-analyses are relevant to a small portion of today’s population. Guo et al. [10] and Schwingshackl et al. [3] consider 1 serving of potatoes as either 80 g or 150 g, respectively, and link an additional serving to a 10% or 18% increase in risk of T2DM. For reference, the USDA Food Patterns Equivalents Database considers a 1 cup equivalent (1 serving) equal to 155 g of a boiled white potato, 120 g of a roasted/baked white potato, or 56 g of potato chips [42]. Muraki et al. [12] report that approximately 2%–4% of cohorts consumed 1 or more servings of potato (total intake) per day, 33%–36% consumed 0.71–0.86 servings per day, and the remaining ∼60% consumed <0.57 servings per day. Thus, the risk Schwingshackl et al. [3] reports from an additional 150 g of potatoes per day, an amount much higher than the intake of the average American consumer, may be relevant to <5% of the population. In contrast, the USDA Food Pattern Equivalents Database [43] reports that 60- to 69-y-old males are the highest potato-consuming demographic, consuming a mean of 0.50 cup equivalents per day (years 2017–2018), or ≤77.5 g/d of boiled white potatoes. Alternatively, 2009–2010 NHANES data demonstrate that adults ≥19 y of age consume ∼39.2 g/d of white potatoes [41], rising to ∼141 g/d for “white potato consumers,” who comprise 28% of the population.

Simply put, routine intakes above 1 serving per day are rare in the United States population, with most demographics consuming half a serving or less per day. Although the meta-analyses suggest that an additional serving (or partial servings) of potato per day linearly increases the risk of T2DM, the majority of the population consumes <1 potato per day. Lastly, the raw data plotted by Guo et al. [10] demonstrate that total potato intake below 100 g/d is more frequently found to reduce or have no impact on the relative risk for T2DM.

## Clinical Trials: Comparisons Matter

Largely absent from the conversation on potato intake and health is an understanding of the biological mechanisms explaining potatoes’ impact on health other than the GI. By nature, the design of most randomized clinical trials (RCTs) cannot prove or disprove changes in disease risk, but they should provide data to support a physiologic mechanism. Data from prospective cohorts suggests that replacing potato intake with whole grains can reduce the risk of T2DM [12]. This observation illustrates that the comparator is critical to understanding RCT results. We hypothesize that for clinical trials, the quality of the carbohydrate food comparator used primarily determines the effect of potato intake. When compared with foods with higher carbohydrate quality scores, potatoes appear “unhealthy.” Compared with foods with equivalent or lower carbohydrate quality scores, potatoes appear “healthy” or “neutral.” Unless specified otherwise, the results discussed in the studies below were statistically significant according to the methods of each article.

A handful of clinical trials [17,[44], [45], [46]] on potato intake compared potatoes with foods high in refined grains with low CFQS-4 and CFQS-5 scores [2], such as white rice, bagels, pasta, and bread. Potato intake appears to have a similar impact on glucose metabolism compared with other refined grains, with preliminary data showing supportive effects on gut health while improving diet quality [17,[44], [45], [46]]. A 1984 study [17] provided early evidence comparing potato intake with refined grain (as a comparison of 2 carbohydrate-rich sources). Andersén et al. [17] compared consumption of potato as a main carbohydrate source with that of isocaloric rice supplemented with lean meat (to match protein between groups) in a 4-wk randomized crossover trial in patients with adult-onset T2DM. Andersén et al. [17] noted equivalent blood glucose, glycated glucose, and HDL and LDL cholesterol but higher VLDL triglyceride and VLDL cholesterol in subjects with T2DM consuming potato. In a randomized, crossover, acute feeding trial in overweight/obese adults with T2DM, compared with isocaloric intake of basmati rice, intake of 3 different preparations of boiled, roasted, or boiled then cooled potatoes showed no significant impact on postprandial triglycerides, glucose, or insulin, except for the finding that boiled and cooled potatoes increased postprandial insulin compared with basmati rice [44]. Dietary analysis showed the GI of the control meal with basmati rice was 54, compared with 83, 76, and 76 for experimental meals containing roasted, boiled, or boiled then cooled potatoes, respectively [44]. All forms of potato resulted in lower nocturnal glucose than the control group. No changes in subjective satiety or physical activity were observed between groups. Devlin et al. [44] ultimately posited that isocaloric intake of potatoes did not elicit an unfavorable postprandial glucose response or nocturnal glycemic control compared with isocaloric intake of basmati rice, despite potatoes’ higher GI. Compared with bagels, isocaloric consumption of potatoes over 2 wk in adults with metabolic syndrome reduced postprandial endotoxemia, with equivalent changes in blood pressure, fasting glucose, insulin resistance, body mass, and other markers of cardiovascular disease in a randomized, crossover trial [46]. In a 4-wk randomized crossover trial in healthy adults, isocaloric consumption of nonfried potato dishes resulted in equivalent effects on fasting plasma glucose, lipids, lipoproteins, blood pressure, weight, and pulse wave velocity as refined grains (e.g., dishes including rice, couscous, orzo, or bread) while also increasing consumption of fiber, potassium, and the Healthy Eating Index 2015 score [45]. Ultimately, each of the aforementioned trials compared potatoes, which on average have a CFQS-4 score between 2 and 3, with food sources of refined grains, which on average have a CFQS-4 score of 0–2 [2], and found mixed, usually neutral, and in some cases beneficial effects of potato intake.

To contrast, in RCTs comparing potato to other energy-rich foods with high CFQS-4 scores or nutrient density (e.g., legumes, nuts, and whole grains) [2], consumption of potatoes frequently exhibited a “null effect,” sometimes with less optimal but not deleterious impacts on the health endpoints measured [[46], [47], [48], [49]]. After a 30-d parallel-arm RCT in healthy adults, intake of almonds (isocaloric comparison to French fries) led to lower postprandial glucose, insulin, and C-peptide response [47] in a meal-based tolerance test. However, fasting glucose, insulin, and fat mass were unchanged between groups, with those consuming French fries losing statistically significant but minimal weight over the course of the study and minimal weight gain in those consuming almonds [47]. In adults with metabolic syndrome consuming a diet based on the Dietary Guidelines for Americans over 2 wk, intake of chickpeas (matched on carbohydrate and fat, but greater in protein and energy than mashed potato) led to lower postprandial glucose, GLP-1, C-peptide, and less hunger and greater fullness [46]. In an 8-wk parallel-arm RCT in healthy adolescents, consumption of potato bread (which had less energy, protein, fat, carbohydrate, and vitamin E, but more fiber, vitamins B1 and B2, ash, sodium, potassium, calcium, and iron) compared with whole-grain bread lowered serum total cholesterol, serum insulin, and serum HDL concentrations while raising systolic blood pressure, with similar impacts on weight loss, diastolic blood pressure, fasting glucose, fasting LDL, fasting triglycerides, and urinary sodium/potassium [48]. In an 8-wk equivalence, parallel-arm RCT in adults with insulin resistance, isocaloric consumption of potatoes compared with beans led to no differences in weight loss, glycemic responses, blood pressure, or lipids, with statistically greater (although similar) reduction in BMI in the group that consumed potato compared with beans [49]. The authors concluded that potatoes and beans were equivalent at reducing insulin resistance and promoting weight loss [49]. Lastly, a recent meta-analysis of RCTs primarily on adults with T2DM has shown that intake of pasta (refined or whole-grain content not specified) stimulates a lesser post-prandial glucose response than potatoes [50]. Overall, these RCTs show that almonds [47], chickpeas [46], and pasta [50], but not beans [49], elicit lesser postprandial glycemic responses than potatoes. Yet overall, each of these foods tended to be similar to potatoes in their longitudinal impacts on weight, body composition, fasting glucose and insulin, fasting lipids, and blood pressure [[46], [47], [48], [49]].

Ultimately, we posit that frequent comparisons to other nutrient-rich foods based solely on their glycemic response in acute feeding trials will continue to strengthen the perception that potatoes are harmful to health, despite inconclusive evidence from RCTs that their consumption has deleterious impacts on cardiometabolic end points. Although consumption of potatoes may be harmful to health if consumed at the complete expense of other high-quality carbohydrates over long periods of time, dietary pattern studies show potatoes are the only high-quality carbohydrate consumed among a wealth of energy-dense foods in Western dietary patterns [19,21,22]. We suggest that other energy-rich and nutrient-poor sources of carbohydrate, such as refined grains, may be more beneficial to limit than potatoes, given refined grain consumption has increased over the past 20 y whereas potato intake has not, and refined grains contribute 6 times the energy to the (American) diet than potatoes [9].

Between forms of potato, those with a low (e.g., baked/boiled and cooled) and high (e.g., cooked but not cooled) GI have largely shown equivalent effects on satiety [44,51,52], with some evidence that low GI potatoes (higher in resistant starch) beneficially lower the postprandial insulin response [52]. However, Devlin et al. [44] showed postprandial blood glucose, insulin, and triglycerides were equivalent between boiled, boiled and cooled, and roasted potatoes in a randomized, crossover, acute feeding trial in subjects with T2DM and overweight/obesity. Andersen et al. [51] demonstrated that Carisma potatoes with a low GI (∼53) are no different from Arizona potatoes with a high GI (∼93) in their impact on satiety and ad libitum meal intake in a randomized, crossover, acute feeding trial in healthy adult men. In an acute, single-dose study, primarily in adult men, the intake of purple majesty potatoes improved plasma antioxidant capacity over 8 h compared with refined starch [53]. The same study detailed a single-arm, noncontrolled, 4-week crossover design in adults with risk of heart disease where consumption of purple majesty potatoes for 4 wk lowered systolic blood pressure, but not weight, fasting plasma glucose, lipids, or hemoglobin A1c [53]. Compared with the consumption of white potatoes, purple and yellow potatoes tend to lower plasma C-reactive protein and 8-hydroxydeoxyguanosine, but not other measures of oxidative stress, such as total antioxidant capacity, protein carbonyl concentrations, or thiobarbituric acid-reactive substances in plasma when consumed over 6 wk in a parallel-arm RCT in healthy adult men [54]. Although intake of purple and yellow potatoes appeared to reduce plasma IL-6 compared with white potatoes, IL-6 was already low at baseline in these groups [54]. Overall, there has been fairly little clinical investigation of the effect of different types/preparations of potato on health.

## Fried Potato Intake Is Confounded with Total Potato Intake

Potatoes are one of the few vegetables often excluded from the vegetable category in nutritional trials. For example, Miller et al. report that Dixon’s and Günther’s Dietary Approaches to Stop Hypertension (DASH) indices consider the potato a vegetable, whereas Fung's DASH index does not [55]. This is echoed in a recent prospective cohort that examined potato intake and T2DM separately from vegetable intake [56]. Pokharel et al. [56] discussed how when potatoes were included as a vegetable in their analysis, the reduction in T2DM risk with vegetable intake lessened, although potato intake was not found to be related to T2DM after adjustment for covariates. Pokharel et al. [56] noted that this association highly depended on the assessed preparation (fried, roasted, or mashed). We are aware of few other vegetables that have been studied as in-depth in different preparations, especially their fried form. As such, potatoes’ impact on health should be considered unique to the form investigated.

It is tempting to conclude that fried forms of potato may be driving any association between potato intake and health. Yet, French fries have similar carbohydrate quality to nonfried forms, with the exception of potato chips, which are vastly more energy dense than most carbohydrate-rich foods (Figure 1). There may not be enough data to conclude that fried forms of potato are the root cause of risk. Dietary pattern studies are usually unclear on the preparation forms of potato measured, given that potato intake is not their focus. Some studies using a posteriori methods to classify dietary patterns separate potatoes from fried forms [4,18] or fast food [20]. Yet, many simply report “potato,” and it is unclear whether fried forms were included [19,[21], [22], [23]]. This starkly contrasts with most meta-analyses (drawing mainly from prospective cohorts) focused on potato intake that describe fried and nonfried forms [3,10,31,57]. Still, there is likely insufficient data to meta-analyze the impact of the preparation method. The most comprehensive meta-analysis on T2DM to date only identified 1 and 2 studies on nonfried and fried potato intake in Western populations, respectively [10]. Furthermore, Guo et al. [10] note that total potato intake does not increase T2DM risk in Eastern populations (RR: 0.94; 95% CI: 0.71, 1.25), but 1 of the 4 studies analyzed did not measure total potato intake [40], only that of French fries and potato chips (and reported intake qualitatively as “routine consumption” or “not routinely consumed”) and reported the greatest RR (2.2; 95% CI: 1.2, 3.9) of any study reviewed by Guo et al. [10]. Given the substantial difference in nutrient quality between potato chips from baked potatoes and even French fries (Figure 1), this misclassification artificially inflated the reported risk of total potato intake, and Eastern populations would have exhibited even lower risk for T2DM if this detail had been rectified. Recent analyses by Pokharel et al. [56] demonstrate the most in-depth assessment of preparation methods, assessing boiled, mashed, roasted, fries/chips, and total potato intake. Such an analysis on other cohort data would be highly valuable. Despite French fries and nonfried potatoes likely having similar carbohydrate quality scores (Figure 1), treating them separately in analyses of dietary patterns may help eliminate the confound between potato intake and general fried food intake (or Western dietary patterns). Doing so with other vegetables would assist in separating the effects of preparation method and behavioral choices of the consumer from the inherent nutrient quality of the food.

In conclusion, decades of reliance on the GI, exaggeration of prospective cohort data, and groupings of potatoes within “Western” dietary patterns have resulted in the perception that the potato should be avoided in the diet. Yet, this notion should be reconsidered in light of its nutritional quality, a more nuanced understanding of the epidemiologic data, emerging clinical evidence, and more comprehensive measures of carbohydrate food quality. Potatoes are one of the highest quality carbohydrate foods consumed in typical Western dietary patterns and may not be the cause of poor diet quality. Consumption rates shown to adversely affect health are high, achieved by a small demographic in the United States, and of lesser concern for most of the population. Clinical trial data support the health effects of the potato as similar to that of legumes and not inferior to refined grains. Therefore, the potato could be considered a healthful and nutrient-dense food choice, rather than a food to be avoided, and included as part of a balanced and varied diet. As public health messaging evolves to reflect a more nuanced understanding of the role of carbohydrates in the diet, it is important to recognize the valuable contribution that potatoes can make to overall diet quality and health.

## Author contributions

The authors’ responsibilities were as follows – SAF, JRM: contributed to conceptualization and writing of the original draft; SAF: review, editing, visualization, and project administration; SAF: had primary responsibility for all content; and both authors: read and approved the final manuscript.

## Conflict of interest

SF reports financial support was provided by FoodMinds, Potatoes USA, and the The Alliance for Potato Research and Education and reports a relationship with Traverse Science that includes: employment and equity or stocks. JM reports a relationship with Traverse Science that includes: employment.

## Funding

Traverse Science received funding from FoodMinds, Potatoes USA, and The Alliance for Potato Research & Education to write the manuscript. Author SAF has ownership in Traverse Science. JRM and SAF are employees of Traverse Science. FoodMinds assisted with conceptualization and editorial review of the perspective but did not contribute to background research, supervision, or writing of the manuscript.

## Data availability

Data for visualizations are available at https://github.com/Traverse-Science/Potatoes-and-carbohydrate-quality.
